# CDK4 as a Prognostic Marker of Hepatocellular Carcinoma and CDK4 Inhibitors as Potential Therapeutics

**DOI:** 10.2174/0109298673279399240102095116

**Published:** 2024-01-12

**Authors:** Fobao Lai, Yingbing Fang, Cong Cheng, Xuejing Zhong, Wanrong Zheng, Shiqian Lan, Quanshui Peng, Xiumei Cai, Tiantian Cao, Chengqian Zhong, Yuzhen Gao

**Affiliations:** 1 Department of Oncology, Longyan First Affiliated Hospital of Fujian Medical University, Longyan, China;; 2 Department of Hepatobiliary Surgery, Longyan First Affiliated Hospital of Fujian Medical University, Longyan, China;; 3 Department of Infectious Disease, Successful Hospital Affiliated to Xiamen University, Xiamen, China;; 4 Department of Science and Education, Longyan First Affiliated Hospital of Fujian Medical University, Longyan, China;; 5 College of Medical Nursing, Minxi Vocational and Technical College, Longyan, China;; 6 Department of Digestive Endoscopy, Longyan First Affiliated Hospital of Fujian Medical University, Longyan, China;; 7 Department of Clinical Laboratory, Sir Run Run Shaw Hospital, Zhejiang University School of Medicine, Hangzhou, Zhejiang, China

**Keywords:** CDK4, CDK4 inhibitor, hepatocellular carcinoma, tumor infiltration, prognosis, T helper cells

## Abstract

**Background:**

The proteins CDK4 and CDK6, which are extremely homologous, control cell cycle entry. For the treatment of breast tumors that include hormone receptors, CDK4 and CDK6 inhibitors have been authorized. The link between CDK4 and liver hepatocellular carcinoma (LIHC), however, has not yet been established.

**Objective:**

The study aimed to explore the link between CDK4 and LIHC and the effect of CDK4 inhibitors on LIHC.

**Methods:**

In this study, we have evaluated CDK4's prognostic relevance in LIHC using data from The Cancer Genome Atlas (TCGA). The relationship between clinical-pathologic features and CDK4 expression has been evaluated using the Kruskal-Wallis test, the Wilcoxon signed-rank test, and logistic regression. We have analyzed CDK4 and factors related to the prognosis of HCC using the Kaplan-Meier technique and multivariate Cox regression. Gene set enrichment analysis (GSEA) identified CDK4-related critical pathways. To investigate the connections between CDK4 and cancer immune infiltrates, TCGA data were employed in single-sample gene set enrichment analysis (ssGSEA). For functional validation, CDK4 was chosen since it can be inhibited by recognized CDK4/6-inhibitors (*e.g.*, abemaciclib).

**Results:**

Poorer overall and disease-specific outcomes were linked to high CDK4 expression in HCC patients. GSEA suggested that CDK4 and immune response are closely connected. The amount of Th2 cells infiltrating was positively correlated with CDK4 expression, while the amount of cytotoxic cells infiltrating was negatively correlated, according to ssGSEA. Both *in vitro* and *in vivo*, the anti-tumor efficacy of CDK4 inhibitor has been found to be superior to that of sorafenib.

**Conclusion:**

This study suggests a relationship between CDK4 and immune infiltration and prognosis in HCC. Additionally, a CDK4 inhibitor may have anti-tumor properties against hepatocellular cancer.

## 1 INTRODUCTION

According to the estimates for global cancer incidence, mortality, and prevalence (GLOBOCAN) 2020, hepatocellular carcinoma (HCC) is the third most frequent cancer and the sixth most common cause of cancer-related deaths. The early asymptomatic nature of HCC makes early diagnosis difficult, and it is very resistant to standard chemo- and radiation; thus, the prognosis is still dismal [1]. Sorafenib, a multikinase inhibitor, is the first approved targeted treatment for HCC patients. RAF-kinase, PDGF, and VEGF receptors are all inhibited by sorafenib [2]. Cell division and proliferation are decreased when the Raf signaling cascade (MAPK/ERK pathway) is blocked by Raf kinase inhibition [3]. Tumor angiogenesis and cell proliferation are suppressed by blocking signal transduction at the PDGF receptor (PDGFR, VEGFR-1, VEGFR-2, and VEGFR-3), which also uses the Raf signaling cascade [4-6]. With the approval of new first- and second-line agents, as well as the acceptance of immune checkpoint inhibitor-based therapies as the standard of care in recent years, the treatment landscape of advanced hepatocellular carcinoma has become more diverse than ever [7-9]. However, the overall survival period is still not ideal, and new drugs must be developed further.

Serine/threonine protein kinases called CDKs are activated when regulatory cyclins attach to them, and in other situations, when different protein kinases phosphorylate them. Because they are important in regulating the cell cycle, CDK1, CDK2, CDK4, and CDK6 have been proposed as potential targets for cancer treatment [10, 11]. Both CDK4 and CDK6 (CDK4/6) are cyclin-dependent serine-threonine kinases that interact with D-type cyclins to phosphorylate the RB tumor suppressor protein in response to mitogenic or pro-proliferative stimuli [12]. This phosphorylation triggers RB's release from E2F transcription factors, allowing E2F to activate the transcription of the genes necessary for the advancement of the G1-S phase cell cycle, and ultimately cellular proliferation. The constitutive activation of cyclin D-CDK4/6 is the main mechanism behind carcinogenesis in several cancer types [13].

Although the present investigations of the capabilities of CDK inhibitors in HCC are in the preclinical stage, there is evidence that CDK inhibitors alone or in conjunction with other chemotherapeutic drugs are useful in the treatment of cancer [13]. The FDA has authorized the use of abemaciclib, a selective cyclin-dependent kinase (CDK) 4 and CDK6 inhibitor, as a treatment for breast cancer [14]. Additionally, HCC and CDK overexpressions were discovered to be related, which supports the idea that this novel agent would work well against HCC [15].

## 2 MATERIALS AND METHODS

### 2.1 Preprocessing and Data Collection

The Cancer Genome Atlas (TCGA) database was utilized to collect RNA-Seq data for 374 HCC patients as well as 50 normal tissues on gene expression, related patients' clinical data, and immune system infiltrates for hepatocellular carcinoma (HCC) patients [16, 17]. Then, utilizing the TCGA's publication standards (https://www.cancer.gov/about-ci/organization/ccg/research/structural-genomics/tcga/using-tcga), we transformed RNAseq data from FPKM to TPM while retaining clinical and RNAseq data.

### 2.2 Immunohistochemistry (IHC) Staining

IHC pictures of CDK4 protein expression in tissues of healthy liver and LIHC were retrieved from the Human Protein Atlas (HPA) (http://www.proteinatlas. org/) [18] and examined to assess variations in CDK4 expression at the protein level.

### 2.3 Differentially Expressed CDK4 Analysis

The expression data (HTseq-Counts) were divided into high and low expression groups based on the median CDK4 expression level and then evaluated using an unpaired Student’s t-test within the DESeq2 R package (3.6.3) [19]. |logFC|<1.5 and adjusted *p*<0.05 were set as the cut-off criteria for the differentially expressed genes (DEGs).

Protein expression analysis was performed on the Clinical Proteomic Tumor Analysis Consortium (CPTAC) dataset using the UALCAN tool (http://ualcan. path.uab.edu/analysisprot.html) [20]. The levels of CDK4 total protein and phosphoprotein expression in primary and normal tissues were evaluated.

### 2.4 Enrichment Analysis

The ClusterProfiler package in R (3.6.3) was used to carry out the functional enrichment analysis for gene ontologies (GO) and the gene set enrichment analysis (GSEA) [21]. The DEGs between high and low CDK4 expression levels were studied. GO analysis includes cellular components (CC), molecular functions (MF), and biological processes (BP). GSEA is a computational tool for detecting if a previously determined set of genes has harmonious variations in two biological states as well as statistical significance [22, 23]. The normalized enrichment score (NES) and adjusted *p*-value were also used to sort the enriched pathways in each phenotype. C2.Cp.v7.2.symbols.gmt (curated) was chosen as the KEGG pathway's reference gene set; gene ontology's C5.all.v7.2 symbols.gmt was chosen as the reference gene set for GO terms [24]. Gene sets with a high rate of false discovery (FDR) were also chosen.

### 2.5 Immune Infiltration Analysis

The GSVA package in R was used to execute ssGSEA in order to extensively examine the immune infiltrates of CDK4 in the research that has been published and investigated the association between CDK4 and the distinctive genes of 24 different immune cell populations [25]. The immunocyte infiltration between the groups with high as well as low CDK4 expression was examined using both the Wilcoxon rank-sum test and the Spearman correlation.

### 2.6 Protein-protein Interaction Network

The search tool for the retrieval of interacting genes database (http://string-db.org) and the Cytoscape software (version 3.7.2) [26, 27] were utilized to evaluate both the functional interaction between proteins and the protein-protein interaction (PPI) network of co-regulated DEGs [28]. In our investigation, 0.7 was the combined score threshold for interaction. Each protein relationship pair in the database achieved a thorough score ranging from 0 to 1. The higher the overall score, the more reliable the PPI relationship was considered.

### 2.7 Cell Lines

HCC cell lines, Huh7 and HepG2, were purchased from the Cell Bank of the Type Culture Collection of the Chinese Academy of Sciences. All cells were routinely cultured in DMEM (Invitrogen) supplemented with 10% fetal bovine serum (FBS; Life Technologies, New York, USA) in a humidified incubator containing 5% CO_2_ at 37°C.

### 2.8 Subcutaneous Tumor Models

The growth inhibitory activity of abemaciclib (50 mg/kg/day, po) and sorafenib (30 mg/kg/day, po) was assessed on the HCC cell line (Huh7). The IC_50_ value of the drugs was determined prior to evaluating their growth inhibitory activity. Cell viability was determined after three days of incubation with the CCK-8 reagent [29, 30]. The xenograft subcutaneous cell line models were prepared as follows:

The human HCC cell line (Huh-7) was grown in Dulbecco’s modified Eagle’s medium (DMEM), which was supplemented with 10% fetal bovine serum (FBS) and 100 units/mL penicillin/streptomycin. The combination was incubated in standard conditions (at 37°C in 5% CO_2_).

A total of 18 4-6-week-old female nude mice were divided into control (n = 7), sorafenib (n = 7), and abemaciclib (n = 7) groups. Female nude mice aged 4-6 weeks were used to create xenograft tumor models. Huh7 cells were subcutaneously injected into the flank of each nude mouse [29]. The mice were kept in pathogen-free conditions (at 22°C, 40-50% humidity, in a 12-hour light/dark cycle). The width and length of subcutaneous tumor volumes were measured from the sixth day, every 5-6 days, until 22 days. Tumor volume was measured by multiplying tumor length by half of the square width of the tumor [31, 32]. Tumors were dissected and weighted after sorafenib and abemaciclib administration. Subcutaneous tumors were removed from the nude mice after 22 days of sacrificing the mice. The tumor weight and body weight of the mice were then recorded.

### 2.9 Western Blot Analysis

Huh7 and HepG2 cell lines were exposed to abemaciclib at concentrations of 0, 0.1, 0.3, 1.0, 3.0, and 10.0 µM. Total cell line protein was extracted using RIPA lysis buffer and PMSF (Thermo Scientific) according to the manufacturers’ instructions. After centrifuging at 13,000 g for 15 min, the supernatant was extracted for further study. Western blots were performed using specific anti-CDK4 (1:1000, ab243872) and anti- GAPDH (1:10,000, ab181602) bodies. Secondary antibodies and CA were purchased from Cell Signaling Technology. The images were captured using the Gel Dox XR system (Bio-Rad, Philadelphia, PA). The experiment was repeated three times independently.

### 2.10 Validation Analysis

Additionally, RNAseq datasets that were retrieved from the GEO database (http://www.ncbi.nlm.nih.gov/geo) were examined to compare the distinct CDK4 expressions between non-tumor tissue and HCC. According to the Kaplan-Meier (K-M) plotter (http://kmplot.com/analysis/index.php?p=service&cancer=liverrnaseq), 54 K genes can be evaluated for their impact on survival in 21 different cancer types. The databases' sources include TCGA, EGA, and GEO. The main objective of the tool is the discovery and validation of survival biomarkers using meta-analyses. To examine the correlation between CDK4 expression and the days of HCC patients' survival, which were represented in K-M survival plots [33], CDK4 was entered into the K-M plotter. Statistical significance was determined by the log rank *p*-value of 0.05.

### 2.11 Statistical Analysis

To process the statistical information from TCGA, R 3.6.3 was utilized. The expression levels of CDK4 in HCC and the healthy group were compared using the Wilcoxon signed-rank tests and Wilcoxon rank-sum. The association between CDK4 expression and the severity of clinicopathological factors was examined using Welch one-way ANOVA. This was followed by the Bonferroni correction or t-test. Univariate logistic regression, normal and adjusted Pearson’s tests, and the Fisher’s exact test were used to investigate the effect of the clinicopathological factors on CDK4 expression. Additionally, we employed Cox regression analysis, both univariate and multivariate, to investigate the predictive significance of CDK4 expression along with other clinico-pathological variables on overall survival (OS). The effectiveness of CDK4 as a prognostic tool was evaluated using the K-M curve. By using univariate Cox proportional hazard regressions, the individual's hazard risk (HR) for overall survival (OS) and disease-specific survival (DSS) were estimated. Each individual factor's HR was estimated (CI) by measuring the HR with a 95% confidence interval. The pROC package was used to carry out the receiver operating characteristic (ROC) analysis of CDK4. The calculated area under the curve (AUC) value ranging from 0.5 to 1.0 showed a 50%-100% discrimination ability. The CDK4 prediction of the HCC outcome at 1, 3, and 5 years was assessed using the time-dependent analysis of the ROC curve. When two-tailed *p*<0.05, all statistical tests were deemed significant.

## 3 RESULTS

### 3.1 Clinical Characteristics

The pathologic stage, tumor status, gender, T stage, N stage, M stage, residual tumor, histologic grade, Child-Pugh grade, OS event, vascular invasion, age, AFP (ng/ml), and BMI were all included in the 374 HCC patients' clinical data (Table **1**). The current study examined 121 females and 253 males in total. The outcome of the Fisher's exact test revealed a significant correlation between CDK4 and OS event (*p* = 0.023); the chi-square test result revealed CDK4 to be significantly associated with T stage (*p* = 0.019), histologic grade (*p* = 0.004), and tumor status (*p* = 0.031). The Wilcoxon rank-sum test revealed a significant correlation between CDK4 and BMI (*p* = 0.022) and AFP (ng/ml) (*p <*0.001). Other clinicopathologic characteristics did not significantly connect with CDK4 expression.

### 3.2 Differential Expression Analysis of CDK4 in HCC

**Fig. (1):**
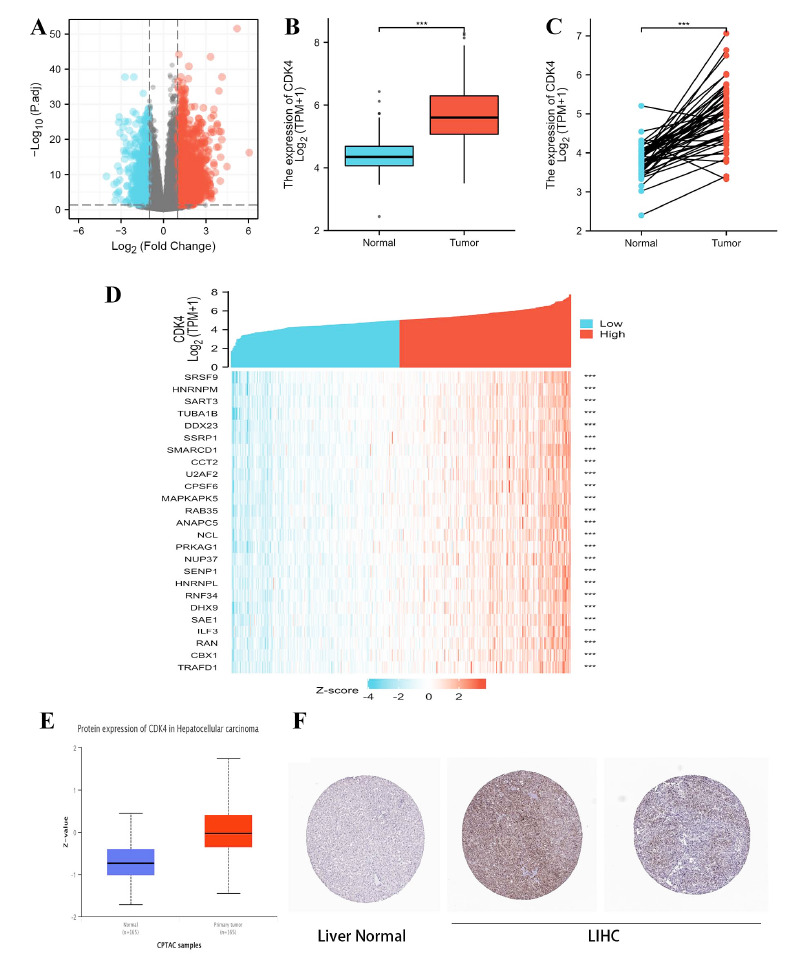
The results of DEG analysis. (**A**) The volcano plot of RNAs with differential expression. (**B**, **C**) The different CDK4 expressions between the LIHC and the normal group. (**D**) The heatmap of the 25 genes with CDK4 correlation. ***p < 0.001. (**E**) CDK4 total protein level in healthy tissue and primary LIHC. CPTAC was used to extract and evaluate protein data. ***p < 0.001. (**F**) Images of CDK4 immunohistochemistry in tumor and normal tissues are compared. Significantly more CDK4 protein has been found to be expressed in LIHC.

Analyzing the TCGA HTSeq count data of CDK4-related genes revealed a total of 951 DEGs. There were 94 downregulated genes and 857 upregulated genes among them. A volcano plot was used to display the DEGs' expressions (Fig. **1A**). Between the normal and LIHC groups, CDK4 expression was considerably higher in tumors than in normal tissue, according to the unpaired and paired different expression analyses (Figs. **1B**, **C**). The heat map in Fig. (**1D**) depicts the relationships between 25 genes and CDK4. Using the large-scale proteome information made accessible by the CPTAC dataset of the National Cancer Institute, we evaluated CDK4 at the protein level in addition to transcription. Fig. (**1E**) shows that LIHC tumor tissue had considerably greater levels of total CDK4 protein expression relative to normal tissue (*p*<0.001). Additionally, we evaluated the IHC results from the HPA database and contrasted them with TCGA's CDK4 gene expression data. Data from these two databases were analyzed, and the findings have been found to be in agreement with one another. While tumor tissues displayed medium or strong CDK4 IHC staining, normal liver tissue displayed negative or moderate staining (Fig. **1F**).

### 3.3 Prognostic Value of CDK4 in LIHC

**Fig. (2):**
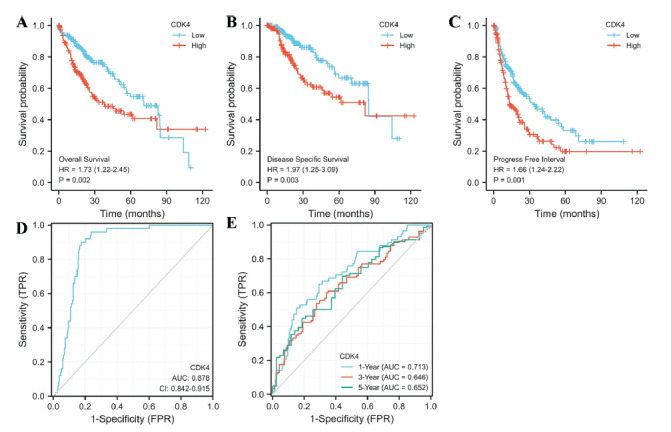
The significance of CDK4 in HCC prognosis. (**A**-**C**) The predictive value of CDK4 in HCC OS, DSS, and PFS. (**D**) Diagnostic ROC curve of CDK4. (**E**) Time-dependent ROC curve of CDK4.

To assess the predictive value of CDK4 in the OS of HCC, the survminer program in R was used to create the K-M survival curve. HCC patients were distributed into high- and low-expression groups based on the median value for CDK4 expression. Poorer OS [HR = 1.73 (1.22-2.45), *p* = 0.00^2^], DSS [HR = 1.97 (1.25-3.09), *p* = 0.00^3^], and PFI [HR = 1.66 (1.24-2.22), *p* = 0.00^1^] were found to be strongly correlated with the high expression group (Figs. **2A**-**C**). The ROC analysis of CDK4 validated the score's diagnostic efficacy (AUC = 0.878, 95% CI: 0.842-0.915) (Fig. **2D**). The ability of CDK4 to predict OS in 1, 2, and 3 years was further assessed using a time-dependent ROC analysis (Fig. **2E**).

**Fig. (3):**
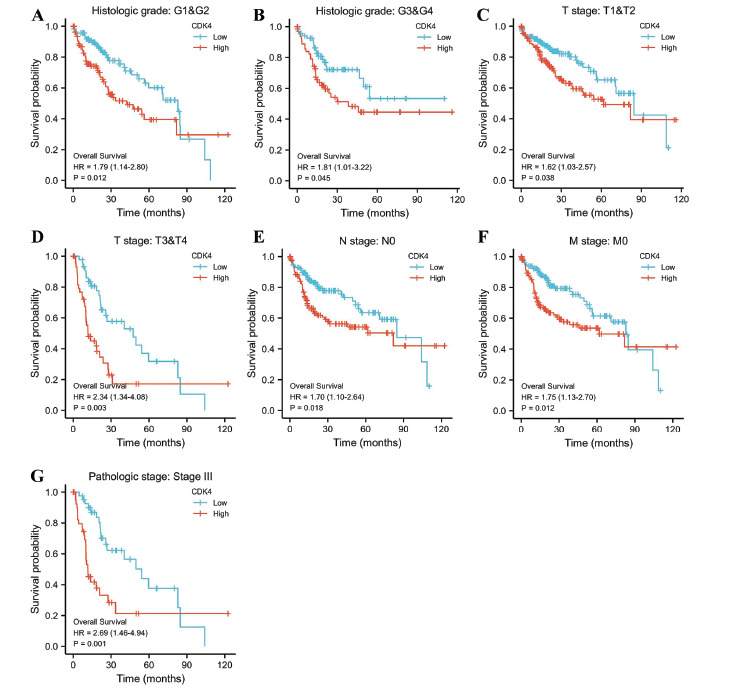
The prognostic value of CDK4 in the different subgroups. (**A**-**G**) Different subgroups showed higher CDK4 expression to be linked to worse OS.

High CDK4 expression was also linked to lower overall survival in the G1 and G2 subgroups of histologic grade [HR = 1.79 (1.14-2.80), *p* = 0.0^12^], G3 and G4 subgroups of histologic grade [HR = 1.81 (1.01-3.22), *p* = 0.0^45^], T1 and T2 subgroups of T stage [HR = 1.62 (1.03-2.57), *p* = 0.0^38^], T3 and T4 subgroups of T stage [HR = 2.34 (1.34-4.08), *p* = 0.00^3^], N0 subgroup of N stage [HR = 1.70 (1.10-2.64), *p* = 0.0^18^], M0 subgroup of M stage [HR = 1.75 (1.13-2.70), *p* = 0.0^12^], and stage III subgroup of pathologic stage [HR = 2.69 (1.46-4.94), p = 0.00^1^] (Figs. **3A**-**G**).

### 3.4 Associations between CDK4 Expression and Clinicopathologic Variables

**Fig. (4):**
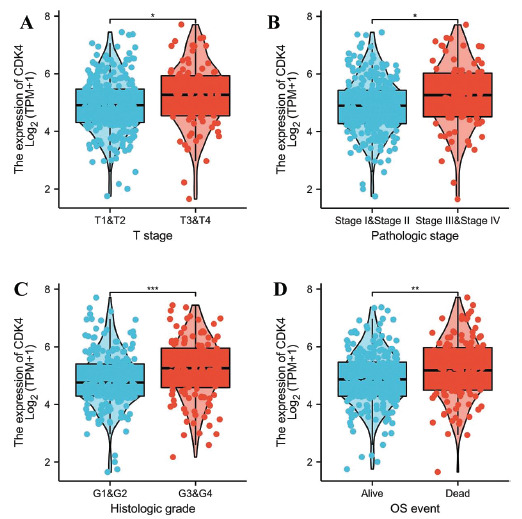
Relationships between various clinicopathologic characteristics and CDK4 expression. (**A**) Relationship between CDK4 expression and HCC T stage, (**B**) Pathologic stage, (**C**) Histologic grade, and (**D**) OS event. *p < 0.05; **p < 0.01; ***p < 0.001.

The Welch’s t-test demonstrated a substantial correlation between CDK4 expression and the T stage and pathologic stage (Figs. **4A**, **B**). The expression of CDK4 was found to be significantly linked with the histologic grade according to the Wilcoxon rank sum test and the OS event according to the t-test (Figs. **4C**, **D**). CDK4 was substantially linked with the T stage (*p* = 0.02), tumor status (*p* = 0.024), histologic grade (*p* = 0.032), and pathologic stage (*p* = 0.055), according to a logit regression analysis (Table **2**).

### 3.5 Immune Infiltration and CDK4 Expression Correlation

The relationship between the ssGSEA score for immune cell infiltration and CDK4 expression levels in TPM format was investigated using the Spearman correlation. Infiltration of Th2 cells has been found to be highly linked with CDK4 expression (Spearman R = 0.480, *p*<0.001) and was higher in the CDK4 high-expression group (*p*<0.001), as shown in Figs. (**5A**, **B**). According to Figs. (**5C**, **D**), the degree of cytotoxic cell infiltration was significantly lower in the CDK4 high-expression group (*p*<0.001) and showed a strongly negative correlation with CDK4 expression (Spearman R = -0.277, *p*<0.001).

**Fig. (5):**
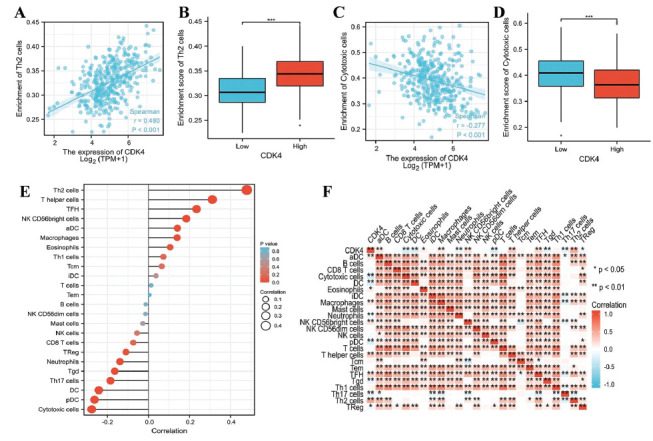
The findings of the research on the link between CDK4 expression and immunological infiltration. (**A**) The association of CDK4 expression with Th2 cells. (**B**) Th2 cell infiltration in various CDK4 expression groups. (**C**) Expression of CDK4 is inversely correlated with cytotoxic cells. (**D**) Cytotoxic cell infiltration in various CDK4 expression groups. (**E**) CDK4 expression and the relative quantity of 24 immune cells are correlated. (**F**) Heatmap of 24 HCC immune infiltrating cells.

Additionally, CDK4 has been found to be positively associated with T helper cells, TFH, NK CD56 bright cells, aDC, macrophages, eosinophils, Th1 cells, and Tcm. A negative connection between CDK4 and pDC, DC, Th17 cells, Tgd, neutrophils, TReg, CD8 T cells, and NK cells has been observed (Fig. **5E**). These findings demonstrated CDK4's critical contribution to immune infiltration in HCC. The degree of correlation between the ratios of the 24 immune cell subpopulations that infiltrate tumors was assessed using a heatmap (Fig. **5F**).

### 3.6 DEGs Functional Enrichment Analysis

**Fig. (6):**
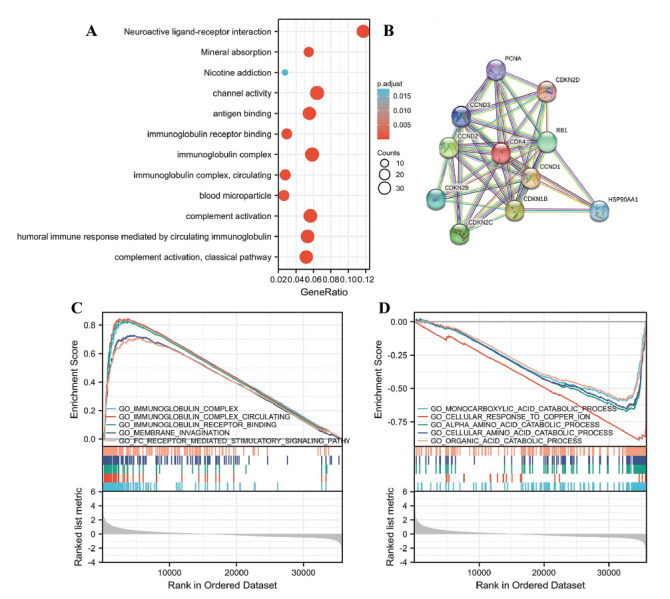
CDK4 enrichment analysis in HCC. (**A**) Enrichment of biological processes involving genes associated with CDK4. (**B**) A CDK4 network with ten possible co-interaction proteins. (**C**, **D**) The GSEA enrichment analysis results.

The results of the GO analysis showed that the DEG-related CDK4 significantly regulated complement activation, classical pathway, humoral immune response mediated by circulating immunoglobulin, complement activation, blood microparticle, immunoglobulin complex circulation, immunoglobulin receptor binding, immunoglobulin complex, antigen binding, channel activity, mineral absorption, and neuroactive ligand-receptor interaction (Fig. **6A**).

Fig. (**6B**) shows the CDK4 network and potential co-expression genes in CDK4-related DEGs.

Immunoglobin complex, immunoglobulin complex circulation, and immunoglobulin complex receptor binding were all positively linked with high levels of CDK4 according to the GSEA analysis in GO terms (Fig. **6C**). High levels of CDK4 were oppositely linked with the activities of the monocarboxylic acid catabolic process, cellular response to copper ion, and alpha-amino acid catabolic process (Fig. **6D**).

### 3.7 CDK4 Inhibitor has an Obvious Killing Effect on HCC Cells

**Fig. (7):**
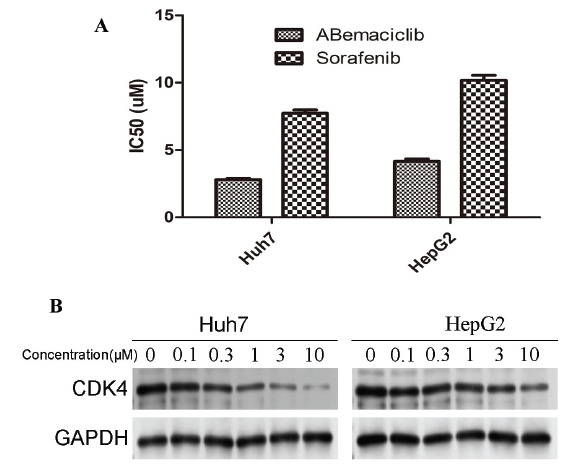
The cytotoxic effect of CDK4 inhibitor on hepatoma cell lines. (**A**) The IC_50_ value of abemaciclib and sorafenib for Huh7 and HepG cells. (**B**) The expression of CDK4 in Huh7 and HepG cells was detected by western blot after treatment with different concentrations of abemaciclib.

The IC_50_ value of abemaciclib for Huh7 and HepG2 cell lines was 4.03 and 2.70 umol/L (uM), respectively. The IC_50_ value of sorafenib for Huh7 and HepG2 was 10.83 and 7.59 uM, respectively (Fig. **7A**). Abemaciclib showed higher efficacy and lower IC_50_ value than Sorafenib. Western blot analysis showed that with the increase in abemaciclib concentration, the expression of CDK4 in hepatoma cell lines decreased more significantly (Fig. **7B**).

### 3.8 The Anti-tumor Effect of CDK4 Inhibitor is Better than that of Sorafenib *In vivo*

**Fig. (8):**
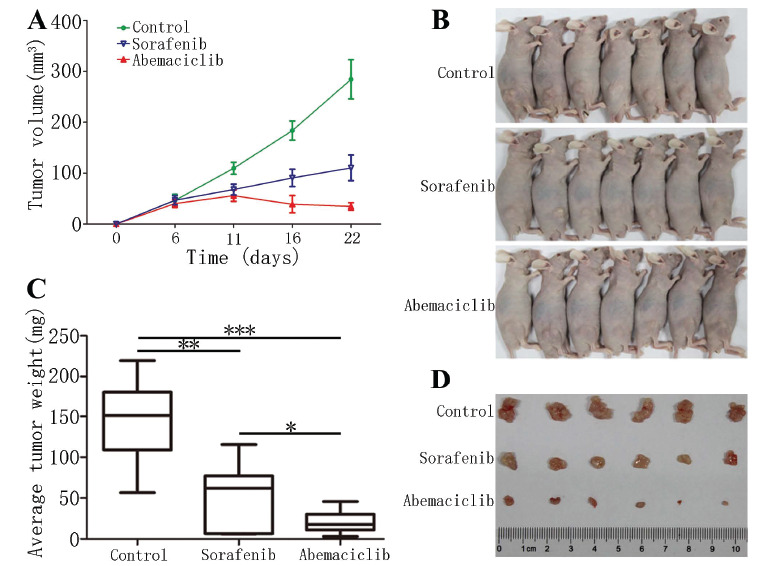
The anti-tumor effect of abemaciclib and sorafenib in vivo. (A) The volume of the subcutaneous tumor was measured every 5-6 days after administration; (**B**-**D**) Tumors were dissected and weighted after abemaciclib (50 mg/kg/D, PO) and sorafenib (30 mg/kg/D, PO) administration.

A lower tumor volume was observed in abemaciclib group compared to sorafenib or control groups (Figs. **8A**, **B**, and **D**). Furthermore, the tumor weight in both sorafenib and abemaciclib groups was lower than the control group (*p <*0.001, *p <*0.01), and the tumor weight in abemaciclib group was lower compared to the sorafenib group(*p <*0.05) (Fig. **8C**); also, there was no significant difference in body weight of mice (Figs. **8** and **S1**).

## 4 DISCUSSION

In HCC patients, CDK4 has been observed to be significantly expressed and linked to a number of advanced clinical characteristics (OS event, T-stage histologic grade, tumor status), implying that CDK4 needs additional clinical validation as a possible prognostic and diagnostic marker. The role of CDK4 in HCC was further examined in GSEA using TCGA data. CDK4 can interact with CCND1, CDKN1B, CCND2, CDKN2C, RB1, CDKN2B, HSP90AA1, CCND3, PCNA, and CDKN2D, according to the PPI network. All these proteins are closely related to cell cycle regulation. According to GSEA, immunoglobulin complex circulation, immunoglobulin complex receptor binding, and immunoglobulin complex are all essential for the development of immune complexes. A possible involvement of CDK4 in the immune response during the carcinogenesis of HCCs has been hypothesized.

To explore the link between CDK4 expression and immune infiltration levels in LIHC, ssGSEA and Spearman correlation were used. Our findings showed that the expression of CDK4 exhibited an important positive association with Th2 cells and T helper cells, as well as a strong-to-moderate correlation with Th1 cells, macrophages, Tfh, aDC, and NK CD56 bright cells. Our results suggest that CDK4 overexpression may cause a shift in the Th1/Th2 balance toward Th2, which is important in HCC metastasis [34]. Th2 cells are responsible for the production of apoptotic factors and influence the recruitment of macrophages and eosinophils into tumors [35, 36], where they produce cytotoxic factors. Moreover, Th2 cells cause IL-5 hypersecretion, which connects Th2 cells and eosinophils in their anti-tumor function [37]. Numerous cancer-related pathways can be activated by IL-4 generated by Th2 cells [38]. The cells have the ability to develop into Th1 and Th2 cells and control humoral immune reactions [39]. The research mentioned above leads us to the conclusion that CDK4 overexpression may cause an immune infiltration in the development of HCC.

Cytotoxic cells, pDC, DC, Th17 cells, Tgd, neutrophils, TReg, CD8 T cells, NK cells, and CDK4 have been found to involve an inverse relationship. NK cells and other cytotoxic cells are essential for anti-tumor immunity. NK cells have a significant role in innate immune defense against malignancy [40]. Through the differentiation of cytotoxic T cells, CD8^+^ T cells exhibit cytotoxic activity against tumor cells [41]. DCs, notably pDC, play a crucial role in the immune system's ability to fight cancer [42]. IFN-I generated by pDC exhibits effective anticancer properties [43]. Th17 cells, which are crucial for tumor immunity and predict a poor prognosis in LIHC, were tightly connected to neutrophils [44, 45]. The development of LIHC may be facilitated by the decreased activity of those immune cells. Since CDK4 and those prognostic indications were related, CDK4 was inferred as a potent prognostic biomarker in HCC.

Furthermore, it was discovered that the CDK4 inhibitor may effectively decrease the expression of CDK4 in hepatoma cell lines and display greater tumor cytotoxicity and a lower IC_50_ value than sorafenib. Additionally, CDK4 inhibitors can significantly reduce the tumor size and weight of transplanted hepatocellular carcinoma in nude mice.

Overall, the research presented here provides a thorough analysis of the part CDK4 plays in HCC progression, which can help in understanding the mechanisms underlying HCC. Clinical research is necessary for investigating the relationship between the expression of CDK4 and clinical characteristics, such as the HCC stage and prognostic value, as it may help identify novel markers for tracking the evolution of the tumor, advancing medication development, and enhancing treatment methods.

## CONCLUSION

In summary, there is an inverse correlation between CDK4 and immune infiltration in HCC, and CDK4 is a potent predictive biomarker in HCC. Additionally, inhibiting the expression of CDK4 may result in a more effective anti-cancer effect in HCC both *in vitro* and *in vivo*, providing a new therapeutic target for HCC.

## Figures and Tables

**Table 1:** Demographic and clinicopathological characteristics of patients with hepatocellular carcinoma in the TCGA-LIHC high and low CDK4 expression groups.

**Characteristics**	**Low Expression of CDK4**	**High Expression of CDK4**	***p*-value**
n	187	187	-
T stage, n (%)	-	-	0.019
T1	102 (27.5%)	81 (21.8%)	-
T2	44 (11.9%)	51 (13.7%)	-
T3	36 (9.7%)	44 (11.9%)	-
T4	2 (0.5%)	11 (3%)	-
N stage, n (%)	-	-	0.624
N0	122 (47.3%)	132 (51.2%)	-
N1	1 (0.4%)	3 (1.2%)	-
M stage, n (%)	-	-	0.622
M0	135 (49.6%)	133 (48.9%)	-
M1	1 (0.4%)	3 (1.1%)	-
Pathologic stage, n (%)	-	-	0.178
Stage I	96 (27.4%)	77 (22%)	-
Stage II	43 (12.3%)	44 (12.6%)	-
Stage III	35 (10%)	50 (14.3%)	-
Stage IV	2 (0.6%)	3 (0.9%)	-
Tumor status, n (%)	-	-	0.031
Tumor-free	113 (31.8%)	89 (25.1%)	-
With tumor	67 (18.9%)	86 (24.2%)	-
Gender, n (%)	-	-	0.507
Female	57 (15.2%)	64 (17.1%)	-
Male	130 (34.8%)	123 (32.9%)	-
Residual tumor, n (%)	-	-	0.215
R0	171 (49.6%)	156 (45.2%)	-
R1	6 (1.7%)	11 (3.2%)	-
R2	1 (0.3%)	0 (0%)	-
Histologic grade, n (%)	-	-	0.004
G1	35 (9.5%)	20 (5.4%)	-
G2	98 (26.6%)	80 (21.7%)	-
G3	47 (12.7%)	77 (20.9%)	-
G4	5 (1.4%)	7 (1.9%)	-
Child-Pugh grade, n (%)	-	-	0.205
A	125 (51.9%)	94 (39%)	-
B	9 (3.7%)	12 (5%)	-
C	0 (0%)	1 (0.4%)	-
Vascular invasion, n (%)	-	-	0.491
No	112 (35.2%)	96 (30.2%)	-
Yes	54 (17%)	56 (17.6%)	-
OS event, n (%)	-	-	0.023
Alive	133 (35.6%)	111 (29.7%)	-
Dead	54 (14.4%)	76 (20.3%)	-
Age, median (IQR)	62 (53, 69)	60 (51, 68.75)	0.292
BMI, median (IQR)	25.28 (22.41, 28.9)	23.88 (21.01, 27.88)	0.022
AFP (ng/ml), median (IQR)	7 (3, 43)	38 (7.5, 2068)	< 0.001

**Table 2:** Logistic regression analysis of the correlation between CDK4 expression and clinicopathological characteristics.

**Characteristics**	**Total (N)**	**Odds Ratio (OR)**	***p*-value**
T stage (T2, T3, and T4* vs. *T1)	371	1.628 (1.082-2.458)	0.020
N stage (N1* vs. *N0)	258	2.773 (0.350-56.463)	0.380
M stage (M1* vs. *M0)	272	3.045 (0.384-61.986)	0.338
Pathologic stage (stage II, stage III, and stage IV* vs. *stage I)	350	1.512 (0.993-2.308)	0.055
Tumor status (with tumor* vs. *tumor-free)	355	1.630 (1.069-2.494)	0.024
Gender (male* vs. *female)	374	0.843 (0.545-1.300)	0.439
Age (>60* vs. *<=60)	373	0.781 (0.519-1.173)	0.234
BMI (>25* vs. *<=25)	337	0.653 (0.423-1.003)	0.052
Residual tumor (R1 and R2* vs. *R0)	345	1.723 (0.662-4.784)	0.273
Histologic grade (G2, G3, and G4* vs. *G1)	369	1.913 (1.068-3.512)	0.032
Adjacent hepatic tissue inflammation (mild and severe* vs. *none)	237	1.253 (0.750-2.101)	0.390
AFP (ng/ml) (>400* vs. *<=400)	280	4.490 (2.464-8.524)	<0.001
Child-Pugh grade (B and C* vs. *A)	241	1.921 (0.795-4.834)	0.151
Vascular invasion (yes* vs. *no)	318	1.210 (0.762-1.924)	0.420
Fibrosis Ishak score (1/2, 3/4, and 5/6* vs. *0)	215	1.298 (0.735-2.313)	0.371
Prothrombin time (>4* vs. *<=4)	297	0.882 (0.534-1.450)	0.620
Albumin (g/dl) (>=3.5* vs. *<3.5)	300	0.909 (0.530-1.562)	0.729
Race (Black or African American and White* vs. *Asian)	362	0.887 (0.585-1.343)	0.571
Weight (>70* vs. *<=70)	346	0.700 (0.457-1.069)	0.099
Height (>=170* vs. *< 170)	341	0.834 (0.540-1.284)	0.410

## Data Availability

The authors confirm that the data supporting the findings of this research are available within the article and its supplementary materials.

## LIST OF ABBREVIATIONS

HCC: Hepatocellular Carcinoma
TCGA: The Cancer Genome Atlas
GSEA: Gene Set Enrichment Analysis
CDK: Cyclin-dependent Kinase
DEGs: Differentially Expressed Genes
LIHC: Liver Hepatocellular Carcinoma
ssGSEA: Single-sample Gene Set Enrichment Analysis
IHC: Immunohistochemistry
GO: Gene Ontology
CC: Cellular Component
MF: Molecular Function
BP: Biological Process
NES: Normalized Enrichment Score
CPTAC: Clinical Proteomic Tumor Analysis Consortium
