# Supplementary material for: CDK4 as a Prognostic Marker of Hepatocellular Carcinoma and CDK4 Inhibitors as Potential Therapeutics
Source: Curr Med Chem. 2024 Jan 12;32(2):343–58. doi: 10.2174/0109298673279399240102095116 (PMC11826894; doi:10.2174/0109298673279399240102095116)
Supplement: Supplementary file 1 [file CMC-32-2-343_SD1.pdf]

Supplementary Material

CDK4 as a Prognostic Marker of Hepatocellular Carcinoma and CDK4 Inhibitors as Potential Therapeutics

Fobao Lai[1,#], Yingbing Fang[2,#], Cong Cheng[3,#], Xuejing Zhong[4], Wanrong Zheng[5], Shiqian Lan[6], Quanshui Peng[2], Xiumei Cai[6], Tiantian Cao[1], Chengqian Zhong[6,*] and Yuzhen Gao[7,*]

[1]Department of Oncology, Longyan First Affiliated Hospital of Fujian Medical University, Longyan, China; [2]Department of Hepatobiliary Surgery, Longyan First Affiliated Hospital of Fujian Medical University, Longyan, China; [3]Department of Infectious Disease, Successful Hospital Affiliated to Xiamen University, Xiamen, China; [4]Department of Science and Education, Longyan First Affiliated Hospital of Fujian Medical University, Longyan, China; [5]College of Medical Nursing, Minxi Vocational and Technical College, Longyan, China; [6]Department of Digestive Endoscopy, Longyan First Affiliated Hospital of Fujian Medical University, Longyan, China; [7]Department of Clinical Laboratory, Sir Run Run Shaw Hospital, Zhejiang University School of Medicine, Hangzhou, Zhejiang, China

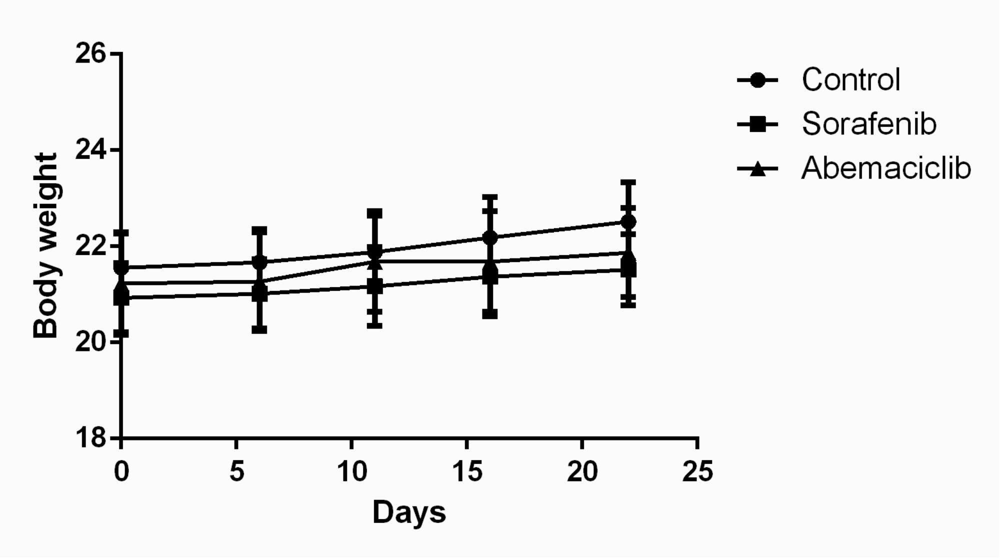

Fig. (S1). The body weight of mice was observed in each group.
